# A novel variant c.7104 + 6T > A of *ABCA12* linked to autosomal recessive congenital ichthyosis verified by minigene splicing assay

**DOI:** 10.3389/fped.2024.1505924

**Published:** 2024-12-19

**Authors:** Linyan Zhu, Rui Zhou, Lianxiao Zhang, Mei Chen, Shengmin Zhang, Xiaxi Huang, Yubo Shi, Huiqing Ding

**Affiliations:** ^1^ Department of Obstetrics and Gynaecology, The First Affiliated Hospital of Ningbo University, Ningbo, Zhejiang, China; ^2^ Department of Ultrasound, The First Affiliated Hospital of Ningbo University, Ningbo, Zhejiang, China

**Keywords:** autosomal recessive congenital ichthyosis (ARCI), *ABCA12* gene, variant, minigene assay, mRNA splicing

## Abstract

**Background:**

Autosomal recessive congenital ichthyosis (ARCI) is a group of genetic skin disorders characterized by abnormal keratinization, leading to significant health issues and reduced quality of life. ARCI encompasses harlequin ichthyosis (HI), congenital ichthyosiform erythroderma (CIE), and lamellar ichthyosis (LI). While all ARCI genes are linked to LI and CIE, HI is specifically associated with severe mutations in the *ABCA12* gene. Milder forms like LI and CIE usually involve at least one non-truncating *ABCA12* variant.

**Methods:**

Whole-exome sequencing (WES) was performed on fetal and parental DNA, and *ABCA12* gene variants were validated by Sanger sequencing. The functional effect of the novel variant c.7104 + 6T > A was evaluated using an *in vitro* minigene system, with splicing analysis conducted via PCR and Sanger sequencing.

**Results:**

A compound heterozygous variation in the ABCA12 gene, comprising c.5784G > A (p.W1928*) and c.7104 + 6T > A, was identified in the fetus, inherited from the father and mother, respectively. According to ACMG guidelines, the c.7104 + 6T > A variant is classified as a Variant of Uncertain Significance (VUS). Computational predictions suggested that this variant affects splicing. A minigene assay further confirmed that the c.7104 + 6T > A variant in ABCA12 leads to two types of aberrant mRNA splicing: a 69-base pair deletion (c.7036_7104del, p.Val2346_Glu2368del) and skipping of Exon 47, both of which result in a premature stop codon and a truncated protein.

**Conclusion:**

In conclusion, this study identified a novel genetic variant, c.7104 + 6T > A in *ABCA12*, as the cause of ARCI in a fetus, thereby enriched the known *ABCA12* mutation spectrum.

## Introduction

Autosomal recessive congenital ichthyosis (ARCI) represents a genetically diverse collection of disorders marked by aberrant keratinization of the skin, which contributes to substantial morbidity and diminished quality of life (1). Within the spectrum of ARCI, mutations in the *ABCA12* gene are notably associated with more severe phenotypes, including harlequin ichthyosis and congenital ichthyosiform erythroderma (2). The prevalence of Autosomal Recessive Congenital Ichthyosis (ARCI) is estimated to range from approximately 1 in 138,000 to 1 in 300,000 live births, with significant variations in incidence observed across different populations, likely attributable to factors such as founder effects (3). Despite advancements in genetic diagnostics, the pathophysiological mechanisms underlying these disorders remain inadequately elucidated, particularly concerning the correlation between specific *ABCA12* mutations and their associated clinical phenotypes (4).

Advanced sequencing technologies have identified numerous variants with the potential to disrupt normal splicing processes (5). The pathogenicity of these variants, especially Non-Canonical Splice-Site Variants, necessitates validation through *in vitro* experiments. Variants located in intronic regions beyond canonical splice sites are frequently overlooked, thereby complicating the diagnosis of rare disorders (6). Consequently, the precise differentiation between splice-affecting and non-splice-affecting variants is of paramount importance. Functional analyses, such as minigene splicing assays, possess significant potential to enhance our understanding of the pathogenicity linked to splicing variants of uncertain significance (7). The minigene splicing assay facilitates the evaluation of the impact of these variants on the splicing process, thereby eliminating the necessity for patient-derived cellular samples.

Pathogenic variants in the *ABCA12* gene cause various forms of autosomal recessive congenital ichthyosis (ARCI), such as severe harlequin ichthyosis (HI) and milder lamellar ichthyosis (LI) and congenital ichthyosiform erythroderma (CIE) (8). Diagnosing ichthyosis can be difficult in patients without a family history of the disorder. Exome sequencing is a swift and dependable method for diagnosing rare Mendelian disorders (9).

In this study, we diagnosed a Chinese fetus with ARCI by identifying specific ABCA12 variants through Whole-exome and Sanger sequencing. The novel c.7104 + 6T > A variant at a noncanonical splice site was classified as VUS. To understand its effects, we used minigene assays to assess the splicing behavior of this variant *in vitro*, providing insights into the disease's molecular pathology. This research is vital for deepening our understanding of splicing abnormalities in genetic disorders. Functional analysis identified the variant's pathogenicity and pathogenesis, broadening the variant spectrum and supporting preimplantation genetic testing for monogenic disorders (PGT-M).

## Materials and methods

### Editorial policies and ethical considerations

The study was approved by the Research Ethics Board of The First Affiliated Hospital of Ningbo University and conducted in accordance with the Declaration of Helsinki. Written consent was obtained from the patient and his legal guardians, where necessary.

### WES and data analysis

WES was conducted as previously described (10, 11). The analysis screened numerous gene variants against databases of pathogenic variations, normal genomes, clinical data on 2,000 genetic diseases, and advanced genetic algorithms.

### Splicing prediction

Splicing prediction of wild-type (WT) and mutant sequences was performed by using RDDC^SC^ (https://rddc.tsinghua-gd.org/), SpliceAI (https://spliceailookup.broadinstitute.org/) and FF (https://www.fruitfly.org/seq_tools/splice.html) with the Default parameters.

### *In vitro* analysis of splice-site mutations of *ABCA12* using the minigene assay

Because the variant NM_173076.3: c.7104 + 6T > A mutation is in intron47, we amplified 1,728-bp gDNA fragments that include Exon46 (110 bp)-Intron46 (302 bp)-Exon47 (142 bp)-Intron47 (1,039 bp)-Exon48 (135 bp) Intron46 (292 bp)-Exon47 (142 bp)-Intron47 (1,039 bp)-Exon48 (135 bp) and inserted them into the eukaryotic expression vector pcDNA3.1 and pcMINI separately to construct recombinant plasmids (pcDNA3.1-*ABCA12*-wt/mut and pcMINI-*ABCA12*-wt/mut). They were digested and verified using gene sequencing. HEK293 and Hela cells were transfected with each recombinant vector using transfection reagent Lipofectamine 2,000 (Invitrogen, Carlsbad, CA, USA) and incubated for 36 h. Total RNA was extracted and reverse-transcribed. The PCR products were identified using 2% agarose gel electrophoresis and verified through sequencing. primer sequences are as followers: pcDNA3.1: forward: 5′-GCTTGGTACCATGTGTTTTGGGCTTCTTGGAGT-3′ and reverse: 5′-TAGAAGGCACAGTCGAGG′, pcMINI:forward:5′-ACTTAAGCTTatgagtgggctttggggtggccggtt-3′ and reverse: 5′-TAGAAGGCACAGTCGAGG-3′.

## Results

### Clinical description

**Figure 1:**
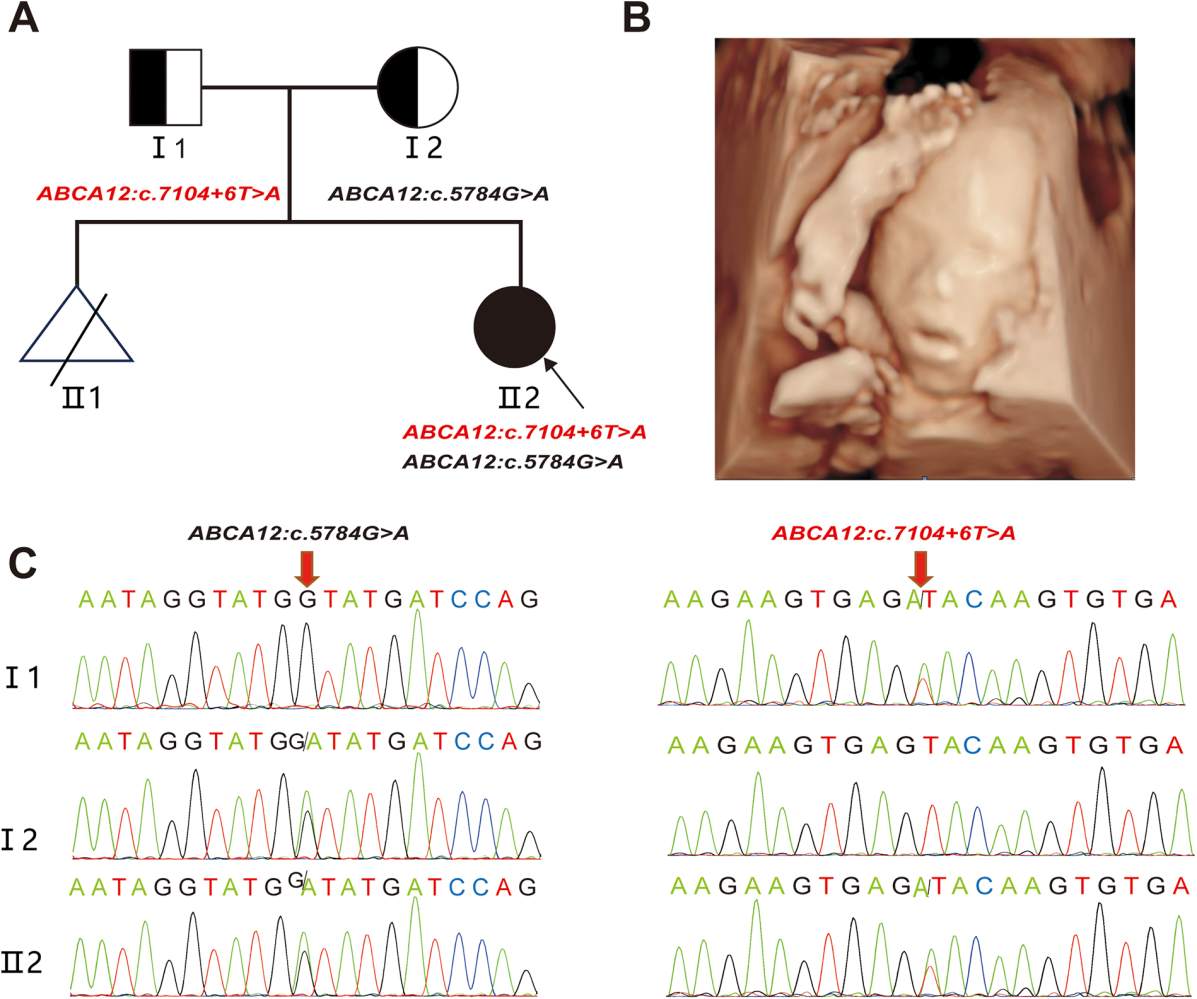
Clinical phenotype **(A)** the pedigree of the family. II2 (the proband) had two compound variants in *ABCA12* gene, which are inherited from the I1(father) and the I2(mother), respectively. **(B)** Fetal ultrasound scan showed: eversion of the lips (eclabium), flexion of fingers. **(C)** Sanger sequencing analysis. The two variants (c.5784 G > A and c.7104 + 6 T > A) were validated by Sanger sequencing. (red arrows indicated the mutation).

A 24—year—old healthy woman was referred to our clinic. Two years ago, her first pregnancy with current husband was terminated at 24th week with fetal heart malformation and no genetic testing was conducted. The woman was already 23^+5^weeks of gestation when she came to our hospital. Ultrasound scans showed that the fetus had small nostrils, a continuously open mouth, clenched fists, and inwardly curled toes (Figures 1AB). To explore the genetic cause, amniocentesis was performed at 24 weeks of gestational age. The compound heterozygous variation in the *ABCA12* gene, consisting of c.5784G > A (p.W1928*) and c.7104 + 6T > A were identified in the fetus and inherited from the father and mother, respectively (Figure 1C). The pregnancy was terminated at 28^+5^th week after the fetal genetic diagnosis and thorough genetic counseling.

This study was approved by the Ethics Committee of The First Affiliated Hospital of Ningbo University and conformed to the Declaration of Helsinki. All participants provided their written informed consents.

### Bioinformatic analysis of variants

**Figure 2:**
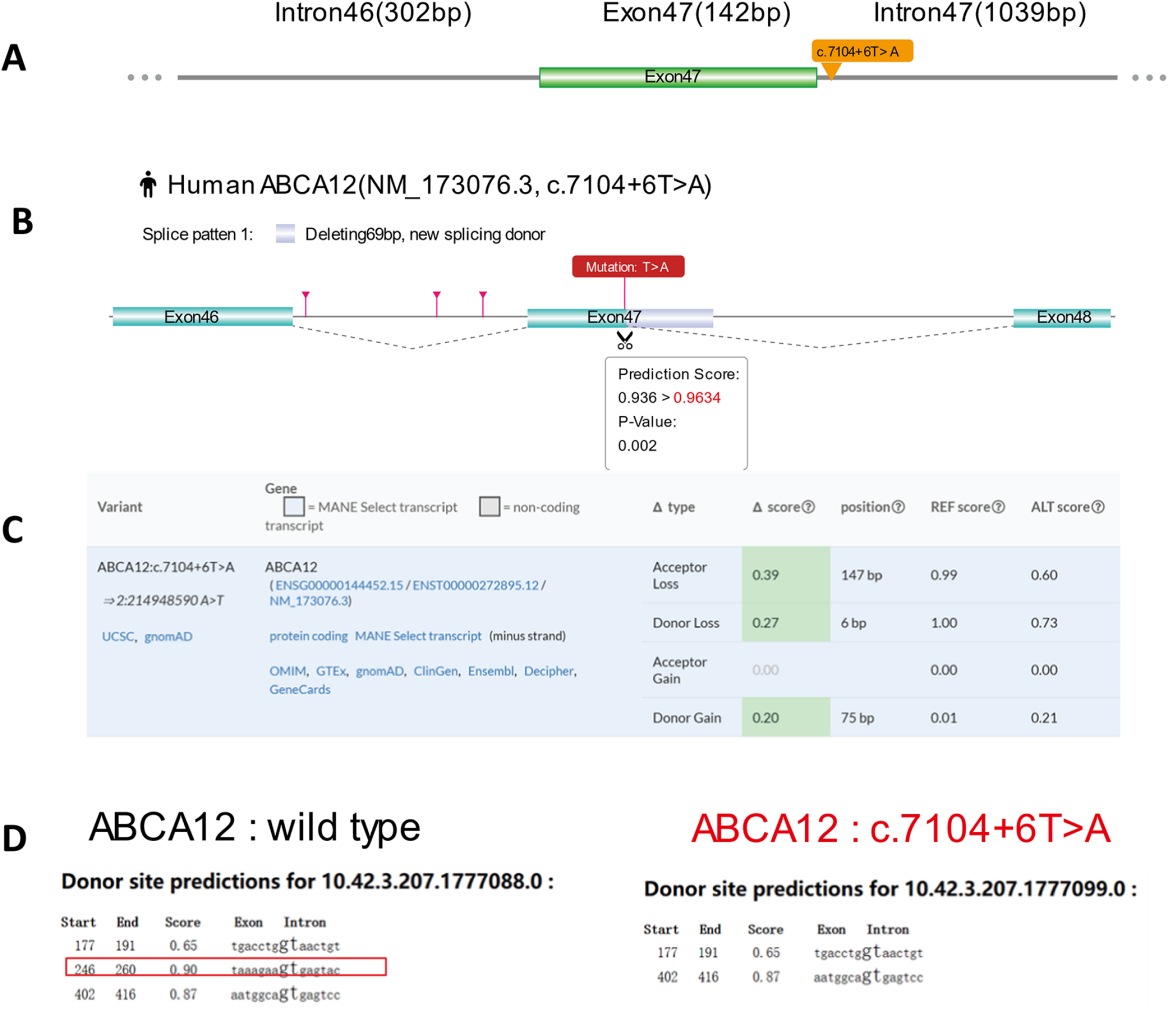
Predictive results of the c.7104 + 6 T > A variant site in splicing. **(A)** Yellow arrow indicates location of the c.7104 + 6 T > A **v**ariant. **(B)** RDDC^SC^ Predicts that c.7104 + 6 T > A variant of *ABCA12* gene affects splicing by deleting 69 bp exon. **(C)** The SpliceAI algorithm shows that the original Acceptor site confidence score decreases by 0.39 after the mutation, the original Donor site confidence score decreases by 0.27, and a new Donor site confidence score of 0.21 may be generated, indicating that the c.7104 + 6 T > A **v**ariant may affect splicing. **(D)** The Predictive result shows that the original donor site is disrupted after the mutation, suggesting that the mutation may affect splicing by using FF.

We performed three bioinformatics databases (RDDC^SC^, SpliceAI and FF) to predict the possible effects of variants on splicing. The c.7104 + 6T > A mutation of *ABCA12* was located at position +6 of exon 47 (Figure 2A).

The RDDC^SC^ (https://rddc.tsinghuagd.org/) and SpliceAI (https://spliceailookup.broadinstitute.org/) analyses suggest that the c.7104 + 6T > A variant may lead to exon deletion (Figure 2B) and affect splicing, as it decreases the original acceptor and donor site confidence scores by 0.39 and 0.27, respectively, and potentially creates a new donor site with a score of 0.21 (Figure 2C). Predictive results confirm that the original donor site is disrupted by using FF (https://www.fruitfly.org/seq_tools/splice.html) (Figure 2D). Three bioinformatics databases all indicate that the c.7104 + 6T > A mutation may affect splicing.

### The c.7104 + 6t > A variant alters *ABCA12* splicing by minigene experimental analysis

**Figure 3:**
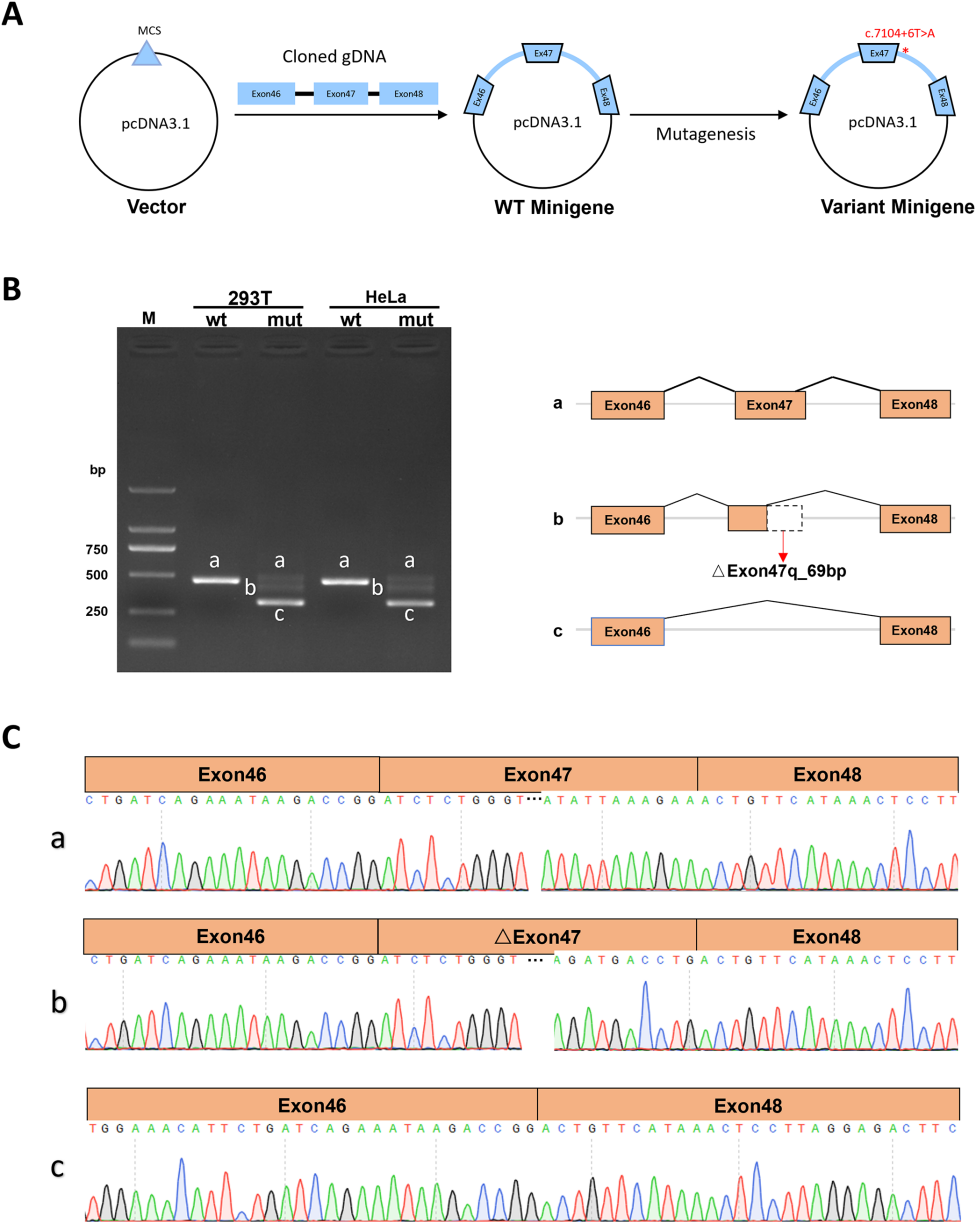
Detection results of the pcDNA3.1 vector. **(A)** Minigene construction strategy diagram. **(B)** RT-PCR transcription analysis of agarose gel electrophoresis image in the pcDNA3.1 vector and splicing schematic diagram, the bands are labeled a, b, c in HeLa and 293 T cells. **(C)** Sequencing results corresponding to the splicing bands.

**Figure 4:**
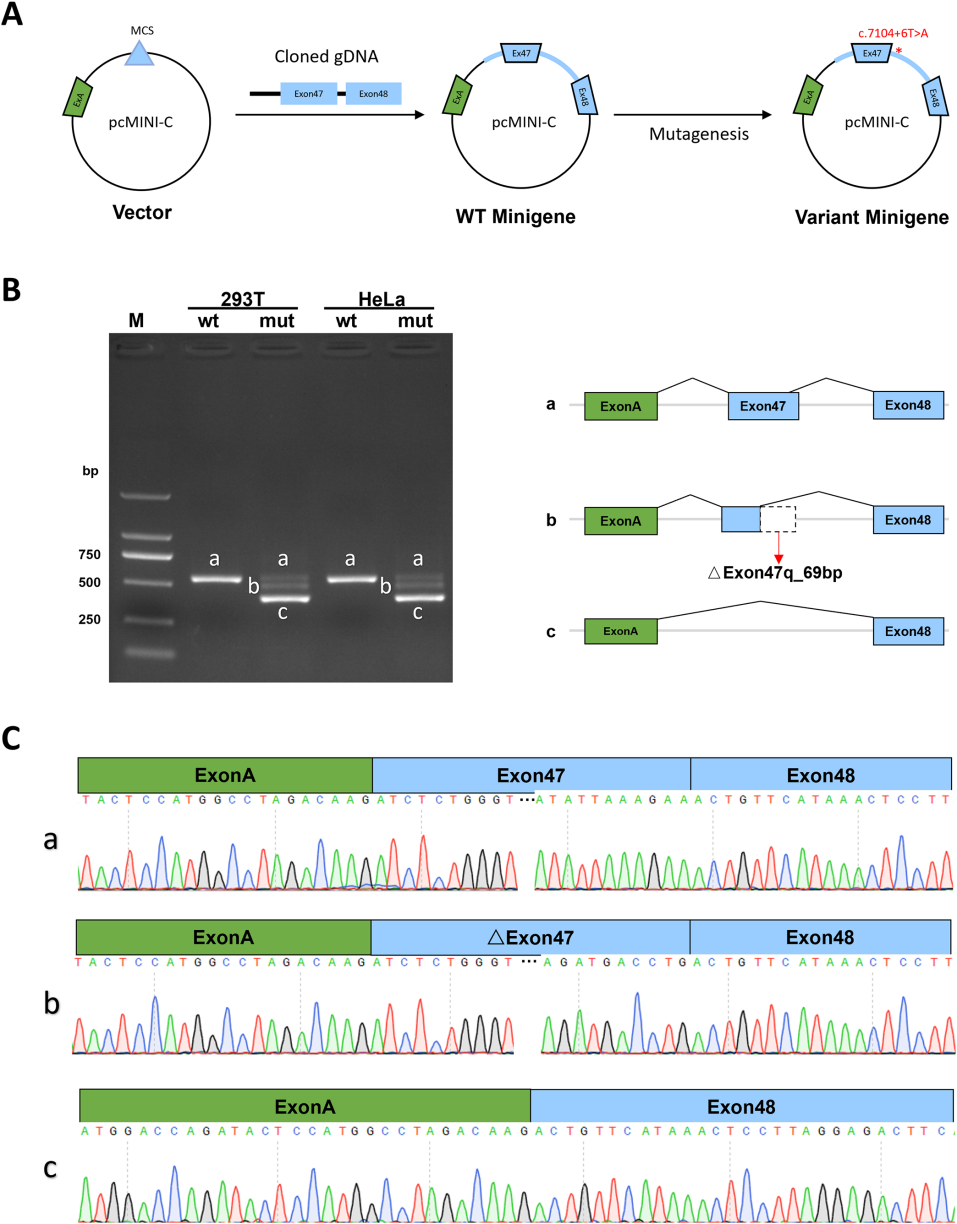
pcMINI-C vector detection results. **(A)** Minigene construction strategy diagram. **(B)** RT-PCR transcription analysis of agarose gel electrophoresis image in the pcMINI-C vector and splicing schematic diagram, the bands are labeled a, b, c in HeLa and 293 T cells. **(C)** Sequencing results corresponding to the splicing bands.

The Minigene *in vitro* experiments show that the variant c.7104 + 6T > A disrupts the normal splicing of the gene mRNA. Additionally, the results from both pcDNA3.1 and pcMINI-C vectors [(Figures 3A, 4A)] are consistent. The minigene of wt showed a splice pattern, and an expected 537-bp-long PCR product ExonA (192 bp)-Exon47 (142 bp)-Exon48 (135 bp) was identified in both HEK293 and Hela cells. In contrast, the minigene of the c.7104 + 6T > A variant of showed three bands, named band a, band b, and band c from largest to smallest (Figures 3B, 4B), and the mutant bands were purified and TA cloned before being sent for Sanger sequencing. Sequencing results showed that the wild type band a was a normal splicing band, with the splicing pattern being ExonA (192 bp)—Exon47 (142 bp)—Exon48 (135 bp); the mutant type band a was also a normal splicing band, with the same splicing pattern; the mutant type band b was an abnormal splicing band, with a 69 bp deletion on the right side of Exon47, and the splicing pattern being ExonA (192 bp)—△Exon47 (73 bp)—Exon48 (135 bp) (Figures 3C, 4C). The 69 bp deletion in Exon47 does not alter the subsequent reading frame, resulting in the loss of 23 amino acids from the protein and potentially producing a shorter protein that is 2,572 amino acids long. The mutant type band c was an abnormal splicing band, with Exon47 skipped, and the splicing pattern being ExonA (192 bp)—Exon48 (135 bp). Skipping of Exon47 alters the subsequent reading frame, creating a premature termination codon (PTC) in Exon48, resulting in a truncated protein that is 2,331 amino acids long.

## Discussion

Autosomal recessive congenital ichthyosis (ARCI) comprises rare genetic disorders marked by abnormal skin keratinization, causing severe scaling and thickening (12). It mainly appears in three forms: lamellar ichthyosis, congenital ichthyosiform erythroderma, and the most severe, harlequin ichthyosis, linked to *ABCA12* gene mutations (13). These mutations impair lipid transport essential for skin barrier function, resulting in significant morbidity and high treatment costs, presenting major challenges (14). Research on autosomal recessive congenital ichthyosis (ARCI) with *ABCA12* gene mutations has enhanced our understanding of genetic skin disorders. Previous studies connected *ABCA12* mutations to ARCI types such as lamellar ichthyosis and congenital ichthyosiform erythroderma (15, 16).

The genotype-phenotype relationship exhibits a robust correlation in autosomal recessive congenital ichthyosis (ARCI), with the *ABCA12* gene frequently implicated in the most severe forms of the disorder. However, fetal ichthyosis often presents with atypical symptoms (17, 18). During the fetal stage, the manifestations of ichthyosis may be relatively mild or nonspecific. For instance, clinical features such as thickened skin, small nostrils, continuously open mouths, and clenched fist postures of the hands may be misinterpreted as normal variations in fetal development or as indicators of other conditions (19). Diagnosing ichthyosis based solely on these symptoms presents significant challenges. Additionally, there is considerable individual variation during the fetal stage (19). Various types of ichthyosis exhibit substantial differences in fetal manifestations; some types may present with minimal or no obvious abnormalities during the fetal stage, thereby complicating the diagnostic process (20). Even within the same type of ichthyosis, symptomatology can vary among different fetuses. In this study, ultrasound examination at 23 weeks gestation identified subtle abnormalities, including small nostrils, persistently open mouths, and continuously clenched hands. Despite the absence of noticeable skin thickening, we considered the potential diagnosis of ichthyosis and consequently conducted whole exome sequencing (WES). The WES results revealed compound heterozygous variations in gene *ABCA12*, inherited from the fetus's parents, which is associated with congenital ichthyosis. Although the genotype-phenotype correlation is consistent, one of the loci in this compound heterozygous variation, the c.7104 + 6T > A variant, is classified as a variant of uncertain significance. Currently, there is insufficient evidence to confirm that this mutation is pathogenic.

We utilized multiple bioinformatics platforms, including RDDC^SC^, SpliceAI and FF, to evaluate the impact of genetic variants on splicing sites. Our analyses consistently indicated that variations in the *ABCA12* gene are associated with abnormal splicing. However, accurately identifying aberrant splicing sites and transcripts solely through bioinformatics tools remains challenging. Experimental verification is crucial to confirm authenticity. The minigene experiment is highly accurate in predicting mutation pathogenicity and splicing effects, and in identifying abnormal exon or intron splicing from single—base substitutions (21, 22). It involves cloning the target genomic fragment, creating a recombinant expression vector, transfecting a cell line, extracting RNA, and Sanger sequencing to assess the impact of variant on mRNA splicing.

To assess the c.7104 + 6 T > A mutation's effect on splicing, *ABCA12* wild-type and variant minigenes were inserted into pcDNA3.1 and pcMINI-C vectors and transfected into HEK293 and HeLa cells. Sequencing revealed three splicing outcomes: normal splicing, a 69 bp deletion in Exon47, and Exon47 skipping. This produced three protein variants: a normal protein, a shorter protein of 2,572 amino acids, and a truncated protein of 2,331 amino acids. Additionally, the c.5784G > A (p.W1928*) nonsense mutation is likely to cause premature termination of polypeptide synthesis. Therefore, it can be clearly concluded that this compound heterozygous variation can cause the congenital ichthyosis.

The aborted fetus is suspected to have ARCI4A due to an *ABCA12* gene variation. This condition usually manifests as a “colloid baby” appearance, progressing to lamellar scales on the body, especially on limb flexors, wrinkled areas, and external genitalia, with taut facial skin. Patients are heat intolerant, often have palmoplantar keratosis, and may exhibit eyelid or lip eversion. The c.7104 + 6 T > A variation likely produces truncated proteins and some normal proteins leading to milder symptoms than harlequin ichthyosis.

Splicing mutations significantly contribute to genetic diseases, and recent studies highlight the potential of engineered U1snRNA to address these mutations (23). Numerous papers have investigated how engineered U1snRNA can positively influence or correct splicing mutations by uniquely interacting with the splicing machinery (24, 25). Future studies should further investigate the potential of engineered U1snRNA in the context of splicing mutations related to our research area, and we should consider this possibility when interpreting and extending our current findings.

To conclude, we identified a splicing variant c.7104 + 6 T > A in gene in a ACRI patientleading to two distinct aberrant splicing effects. Our findings expanded the variant spectrum and provided a strong indication and sufficient basis for PGT-M.

## Data Availability

The datasets for this article are not publicly available due to concerns regarding participant/patient anonymity. Requests to access the datasets should be directed to the corresponding author.
